# Additive effects of coexisting respiratory comorbidities on overall or respiratory mortality in patients with asthma: a national cohort study

**DOI:** 10.1038/s41598-022-12103-w

**Published:** 2022-05-16

**Authors:** Yoomi Yeo, Hyun Lee, Jiin Ryu, Sung Jun Chung, Tai Sun Park, Dong Won Park, Sang-Heon Kim, Tae Hyung Kim, Jang Won Sohn, Ho Joo Yoon, Kyung Hoon Min, Ji-Yong Moon

**Affiliations:** 1 Department of Internal Medicine, Hanyang University College of Medicine, Seoul, South Korea; 2 Biostatistical Consulting and Research Lab, Medical Research Collaborating Center, Hanyang University, Seoul, South Korea; 3 Division of Respiratory and Critical Care Medicine, Department of Internal Medicine, Korea University Guro Hospital, Korea University Medical School, 148, Gurodongro, Guro-gu, 08308 Seoul, South Korea; 4 Division of Pulmonology and Critical Care Medicine, Department of Internal Medicine, Hanyang University Guri Hospital, Hanyang University College of Medicine, 153, Gyeongchun-ro, Guri-si, Gyeonggi-do 11923 South Korea

**Keywords:** Medical research, Epidemiology, Outcomes research

## Abstract

Asthmatic patients are generally considered to have an increased risk of mortality compared with subjects without asthma. However, this issue has been less evaluated using nationally representative data. Moreover, it is unclear whether respiratory comorbidities other than chronic obstructive pulmonary disease (COPD) are associated with increased mortality in asthmatic patients compared with subjects without. Using a nationally representative sample database, we performed a retrospective cohort study of patients with asthma and age-sex-matched control cohort. We estimated the hazard ratio (HR) and stratified the asthma cohort based on respiratory comorbidities. During a median 8.9-year follow-up, the overall mortality rate was higher in the asthma cohort than in the control cohort (p < 0.001). The hazard ratio (HR) for overall mortality in the asthma cohort compared with the control cohort was 1.13. The effects of asthma on overall mortality were more evident in males, patients under medical aid, and subjects with COPD. Respiratory comorbidities were significantly associated with increased risk of overall mortality in asthmatic patients compared with controls (adjusted HRs; 1.48 for COPD, 1.40 for bronchiectasis, 4.08 for lung cancer, and 1.59 for pneumonia). While asthma and lung cancer showed an additive effect only on overall mortality, asthma and other respiratory comorbidities (COPD, pneumonia, and bronchiectasis) had additive effects only on respiratory mortality. Patients with asthma had a higher overall mortality rate compared with subjects without asthma. Respiratory comorbidities showed an additive effect on overall or respiratory mortality in patients with asthma.

## Introduction

Asthma affects 5–10% of the population and contributes to approximately 0.4 million deaths annually worldwide^1^. Asthma causes a high global burden of death and disability and is in the top 20 causes of years of life lived with disability. Although the mortality in asthmatic patients has decreased, asthma still has a sizable health-related burden of life^2,3^.

Asthmatic patients are generally considered to have higher mortality than subjects without asthma^4–6^. In a large population-based matched cohort study, the mortality in asthmatics was higher than in controls^4^. Another study reported similar results using a provincial health database^6^. However, in most countries, it remains inconclusive which factors cause higher mortality in patients with asthma compared with subjects without asthma because of the lack of evaluation of this issue using a nationally representative database^4,6^.

Respiratory conditions including chronic obstructive pulmonary disease (COPD), bronchiectasis, lung cancer, and pneumonia are important comorbidities that affect the natural course of asthma. Coexisting COPD is associated with poor asthma control and increased healthcare use and is an important risk factor for mortality in patients with asthma^4,6^. However, except for COPD, the association of these comorbidities and mortality in patients with asthma has not been well elucidated, although the comorbid conditions have potential deteriorating effects of asthma on mortality. For example, bronchiectasis is associated with the severity of asthma^7^, and asthmatic patients are at increased risk of serious diseases, such as lung cancer^8^ and pneumonia^9^, which can result in increased mortality in patients with asthma.

Thus, this study aimed to compare the mortality rate in patients with and without asthma using a representative nationwide database. We also evaluated the effects of asthma-related respiratory comorbidities on mortality in patients with asthma compared with subjects without asthma.

## Methods

### Study population

In South Korea, almost all Korean citizens are enrolled in the National Health Insurance System (NHIS). The NHIS collects health data from nearly 50 million insured subjects, including admission and outpatient visit records, diagnoses, drug prescriptions, national health examination data, death, and causes of death. The NHIS provides all the above-mentioned information for research purposes. For the present study, we used data from the NHIS-National Sample Cohort (NSC), a specialized data set provided by NHIS that includes a stratified random sample for the total eligible Korean population (approximately 1 million people)^10^. In this study, all methods were performed under the relevant guidelines and regulations.

A total of 738,254 patients 18 years of age or older were identified from January 1, 2005, to December 31, 2006. After excluding 16,995 subjects with malignancy in the previous three years (washout period), 697,281 patients were selected. Among these, 80,356 patients had at least one claim for ICD-10 codes for asthma (J45–46). Among the 80,356 patients with asthma, we excluded patients who had asthma during the three-year washout period (n = 15,069), patients who had only one claim under ICD-10 codes for asthma (n = 28,333), patients who did not receive asthma-related medication (n = 16,592), and patients who died during the baseline period (n = 55). Finally, a total of 19,319 patients with incident asthma was identified (asthma cohort). Next, we performed 1:4 age and sex matching for each asthma patient from the cohort without asthma (control cohort). Patients were followed-up until the date of death or December 31, 2015 (Supplemental Fig. 1).

### Definitions

#### Asthma

Adult asthma was defined based on the following criteria: (1) 18 years of age or older, (2) at least two claims under the 10th revision of the International Statistical Classification of Diseases and Related Health Problems (ICD-10) codes J45–46, and (3) at least one claim for prescription of asthma-related drugs including inhaled or systemic corticosteroids, bronchodilators, leukotriene receptor antagonists, or xanthine derivatives^11,12^. The index event for incident asthma was defined as the first prescription of asthma-related medication with ICD codes J45–46 as a major or minor diagnosis between January 1, 2005, and December 31, 2006, with no diagnosis code more than 3 years before the first index event after the sensitivity test. The baseline period was defined as 12 months before the index date. The follow-up period was from the index date to the date of death or December 31, 2015.

#### Comorbidities

Baseline comorbidities were defined as comorbidities with at least one claim under ICD-10 codes using a major or minor diagnosis during the baseline period as follows: respiratory diseases (J00–J99) including COPD (J43 or J44 except for J43.0), bronchiectasis (J47), and pneumonia (J12–J18); cardiovascular diseases (I00-I99) including hypertension (I10–I15), ischemic heart diseases (I20–I25) including angina (I20) and myocardial infarction (I21), and congestive heart failure (I50); cerebrovascular diseases (I60–I69); endocrine diseases (E00–E90) including diabetes mellitus (E10–E14); gastrointestinal diseases (K00–K93); neurologic diseases (G00–G99); mental and behavioral disorders (F00–F99); and musculoskeletal and connective tissue diseases (M00–M99) including osteoporosis (M80–M82)^13^.

#### Causes of deaths

For the analyses of causes of death, we used mortality data provided by Statistics Korea (an initiative of the Ministry of Strategy and Finance of the Republic of Korea^10^. We evaluated all-cause mortality as well as respiratory disease-related mortality. Respiratory disease-related mortality was defined as deaths under ICD-10 codes for respiratory diseases (J00-J99).

### Statistical analysis

The participants in the control cohort group were identified using 1:4 matching in which the nearest available person for each case was selected as a matched cohor^14^. We compared the baseline characteristics (age, sex, type of insurance, Charlson Comorbidity Index)^15^ using the McNemar test because each asthmatic patient was matched to several non-asthmatic patients. The Kaplan–Meier method was used to estimate survival curves during the follow-up period, and survival was compared among groups using the log-rank test. Hazard ratio (HRs) with 95% confidence interval (CI) for the mortality of patients in the asthma cohort compared with subjects in the control cohort were evaluated using an age- and sex-stratified Cox regression model. To evaluate the effects of asthma-related pulmonary comorbidity (COPD, pneumonia, bronchiectasis, and lung cancer) on mortality in patients with asthma compared with subjects without asthma, the asthma cohort was classified into two groups based on each comorbidity. Next, we performed multivariable Cox regression analyses to evaluate the effects of each respiratory comorbidity on HR for mortality (age and sex were adjusted in Model 1; variables in Model 1 and type of insurance were adjusted in Model 2). To evaluate additive effects of asthma and its respiratory comorbidities on overall and respiratory mortalities, the asthma cohort and the control cohort were classified into two groups based on each comorbidity. Thereafter, we performed multivariable Cox regression analyses (age and sex were adjusted in Model 1; variables in Model 1 and type of insurance were adjusted in Model 2).

All analyses were conducted using SAS 9.4 (SAS Institute, Cary, NC, USA). All tests were two-sided, and p-values < 0.05 were considered statistically significant.

### Ethics approval and consent to participate

This study was approved by the Ethics and Review Board of Hanyang University Guri Hospital (IRB No. 2019-11-018). The requirement of informed consent from the participants was waived by the Ethics and Review Board of Hanyang University Guri Hospital because the NHIS database was constructed after anonymization.

## Results

### Participants

The baseline characteristics of asthma and control cohorts are summarized in Table 1. The mean age of patients in the asthma cohort was 49 years, and 64.1% were female. There were no significant differences regarding age and sex. Regarding the type of insurance, patients in the asthma cohort were more likely to receive medical aid compared with subjects in the control cohort. Comorbidity profiles are summarized in Supplemental Table 1.

**Table 1:** Baseline descriptive characteristics of the study population.

	Total (n = 96,595)	Asthma cohort^a^ (n = 19,319)	Control cohort (n = 77,276)	p-value
Age, years	49 (36–64)	49 (36–64)	49 (36–64)	
**Age group**				> 0.99
Twenties	11,880 (12.3)	2376 (12.3)	9504 (12.3)	
Thirties	19,255 (19.9)	3851 (19.9)	15,404 (19.9)	
Forties	18,370 (19.0)	3674 (19.0)	14,696 (19.0)	
Fifties	15,750 (16.3)	3150 (16.3)	12,600 (16.3)	
Sixties	16,610 (17.2)	3322 (17.2)	13,288 (17.2)	
Seventies	14,730 (15.3)	2946 (15.3)	11,784 (15.3)	
**Sex**				> 0.99
Male	34,720 (35.9)	6944 (35.9)	27,776 (35.9)	
Female	61,875 (64.1)	12,375 (64.1)	49,500 (64.1)	
**Type of insurance**				< 0.01
Self-employed health insurance	39,519 (40.9)	7369 (38.1)	32,150 (41.6)	
Employee health insurance	52,047 (53.9)	10,198 (52.8)	41,849 (54.2)	
Medical aid	5029 (5.2)	1752 (9.1)	3277 (4.2)	

Data are presented as frequency (%) and mean with standard deviation.

COPD, chronic obstructive pulmonary disease.

^a^Asthma-related medication included inhaled corticosteroid (32%), long-acting β2 agonist (19.1%), short-acting β2 agonist (34.8%), and leukotriene receptor antagonist (35.7%).

### Mortality

**Figure 1:**
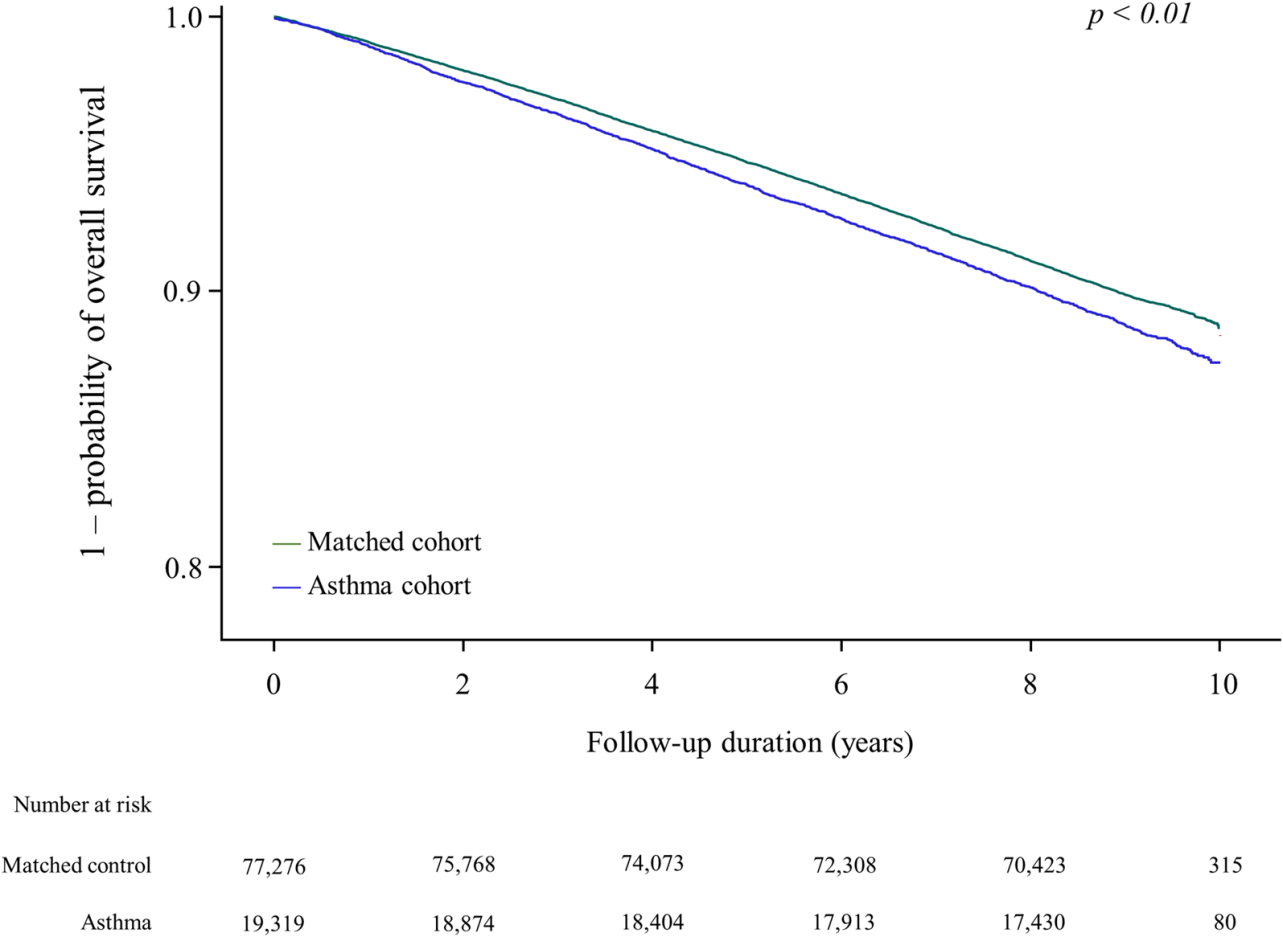
Kaplan–Meier survival plot of time to death in the asthma cohort versus the control cohort.

**Figure 2:**
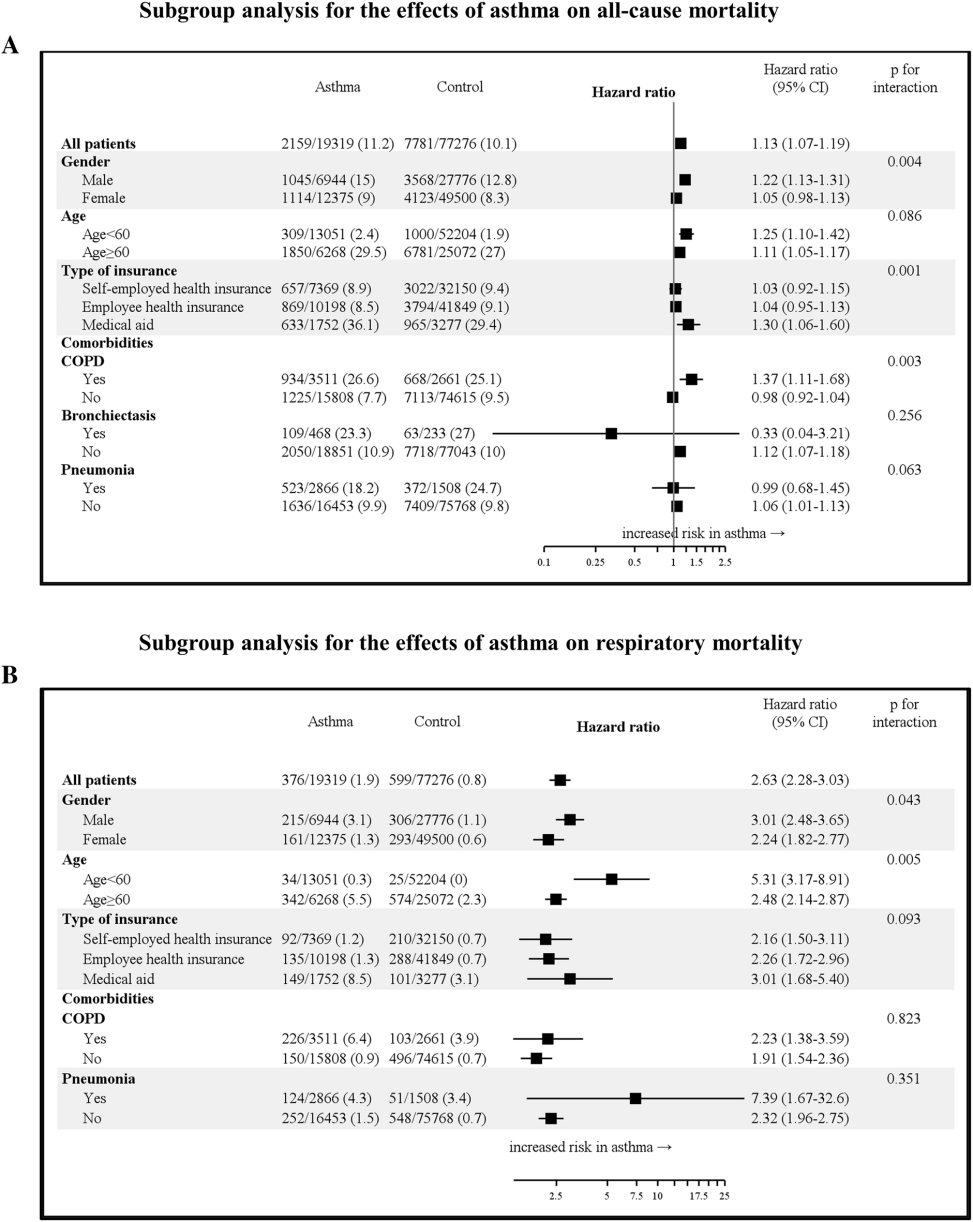
Subgroup analysis for the effects of asthma on mortality. (**A**) all-cause mortality, (**B**) respiratory mortality.

During a median 8.9-year follow-up duration, the overall mortality rate was 1312/100,000 person-years (PY) in the asthma cohort and 1174/100,000 PY in the control cohort (p < 0.01), which is consistent with survival analysis (p < 0.01 for a log-rank test; Fig. 1). Patients in the asthma cohort had a significantly higher all-cause mortality rate than subjects in the control cohort (HR = 1.13, 95% CI = 1.07–1.19). As shown in Fig. 2A, a positive association between asthma and all-cause mortality was observed in both age groups. However, the association was only significant in males, patients who received medical aid, patients with COPD, and patients without bronchiectasis. The association was stronger in males than females (p for interaction = 0.004), in patients who received medical aid than in patients who received other medical insurance (p for interaction = 0.001), and in patients with COPD than in patients without COPD (p for interaction = 0.003).

The adjusted HR (aHR) for respiratory mortality was 2.63 (95% CI = 2.28–3.03). As shown in Fig. 2B, the positive association between asthma and respiratory mortality was consistent in all subgroups. The association was stronger in males than females (p for interaction = 0.043) and in younger patients (age < 60 years) than older patients (age $\ge$ 60 years; p for interaction = 0.005).

### The effects of asthma-related respiratory comorbidities on mortality in the asthma cohort compared with the control cohort

**Figure 3:**
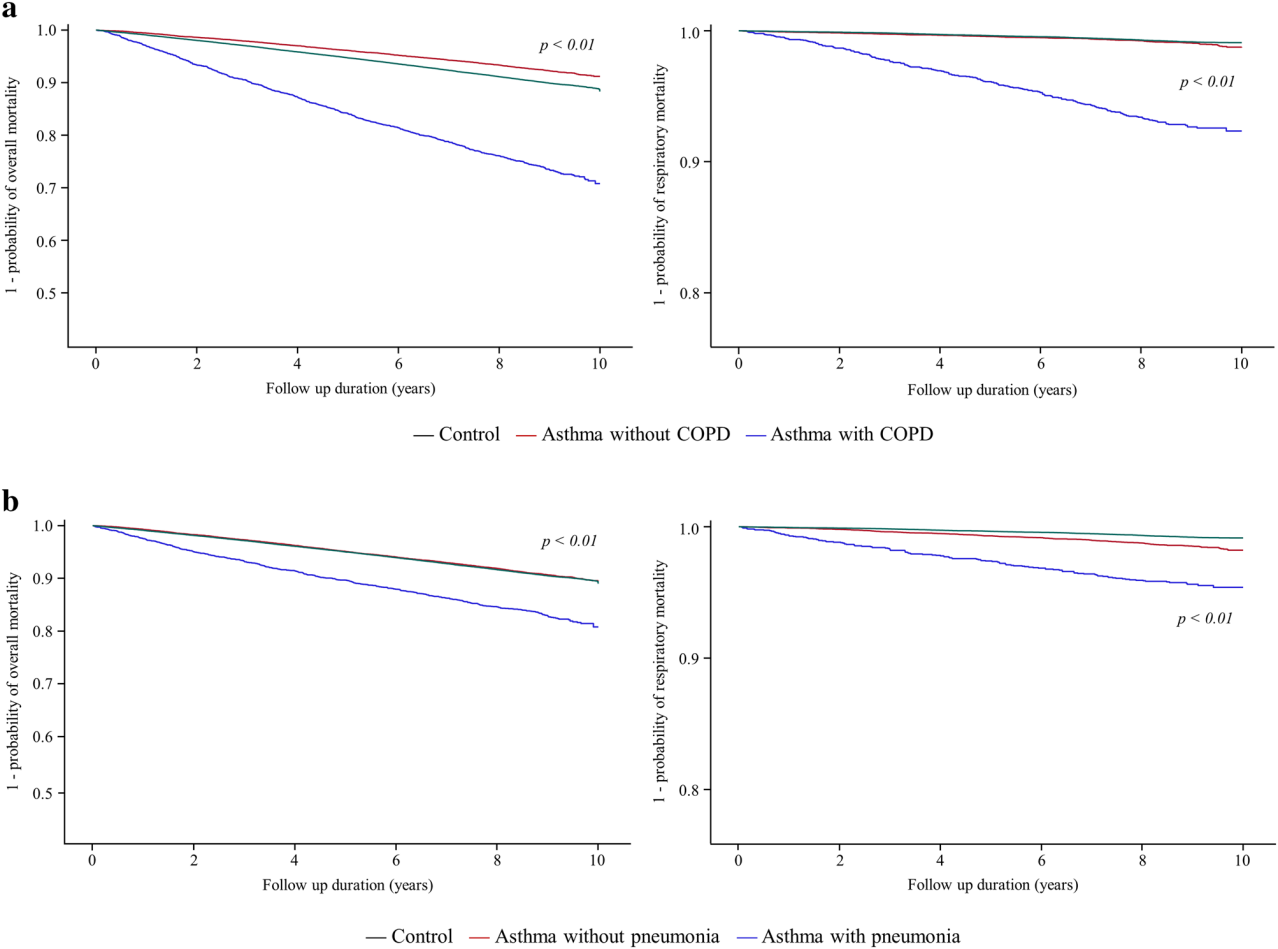

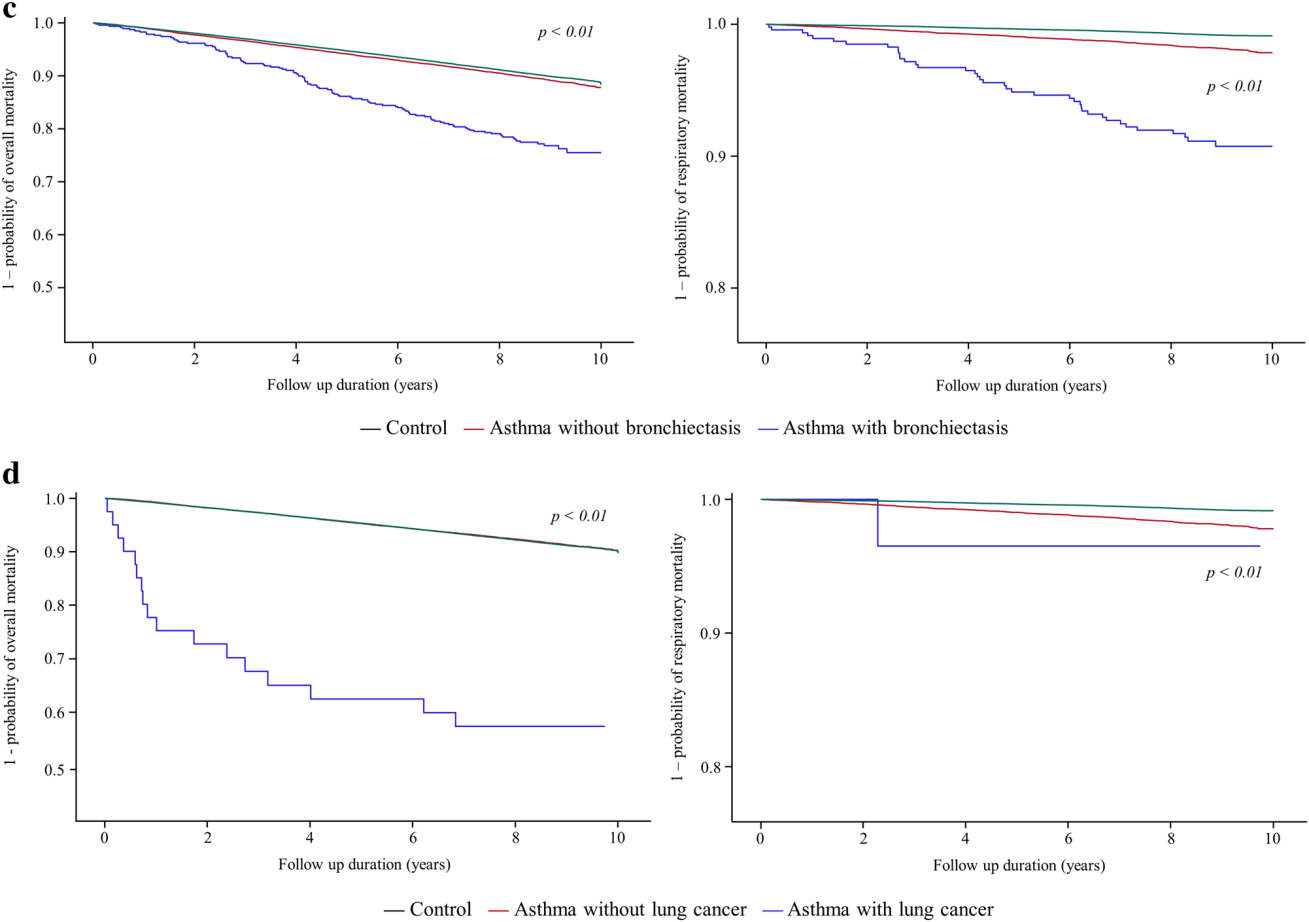
Kaplan–Meier survival plot of time to death in asthmatic patients with comorbidities, asthmatic patients without comorbidities, and subjects without asthma. (**a**) COPD, (**b**) pneumonia, (**c**) bronchiectasis, and (**d**) lung cancer. COPD, chronic obstructive pulmonary disease.

As shown in Table 2, the relative risk of overall mortality in asthmatic patients without COPD (aHR in Model 2 = 0.94, 95% CI = 0.89–1.00) or pneumonia (aHR in Model 2 = 1.02, 95% CI = 0.97–1.08) compared with the controls was not significantly increased; however, asthmatic patients with COPD (aHR in Mode 2 = 1. 46, 95% CI = 1.36–1.56) or pneumonia (aHR in Mode 2 = 1.56, 95% CI = 1.42–1.70) had increased risk of overall mortality compared with the controls, which is in agreement with survival analyses (COPD in Fig. 3a and pneumonia in Fig. 3b).

**Table 2:** The effects of asthma and its coexisting respiratory comorbidities on mortality in patients with asthma compared with subjects without asthma.

	Unadjusted model		Model 1		Model 2	
	All-cause	Respiratory	All-cause	Respiratory	All-cause	Respiratory
	HR (95% CI)	HR (95% CI)	aHR (95% CI)	aHR (95% CI)	aHR (95% CI)	aHR (95% CI)
**COPD**						
Control cohort	Reference	Reference	Reference	Reference	Reference	Reference
Asthma cohort without COPD	0.76 (0.72–0.81)	1.21 (1.01–1.44)	0.95 (0.90–1.01)	1.58 (1.32–1.89)	0.94 (0.89–1.00)	1.53 (1.28–1.84)
Asthma cohort with COPD	2.94 (2.75–3.14)	9.31 (7.99–10.85)	1.49 (1.39–1.59)	4.27 (3.66–4.99)	1.46 (1.36–1.56)	3.95 (3.38–4.61)
**Pneumonia**						
Control cohort	Reference	Reference	Reference	Reference	Reference	Reference
Asthma cohort without pneumonia	0.99 (0.94–1.04)	1.97 (1.70–2.29)	1.03 (0.98–1.09)	2.08 (1.79–2.41)	1.02 (0.97–1.08)	1.99 (1.71–2.30)
Asthma cohort with pneumonia	1.92 (1.75–2.09)	5.92 (4.88–7.19)	1.59 (1.46–1.74)	4.72 (3.89–5.73)	1.56 (1.42–1.70)	4.33 (3.57–5.27)
**Bronchiectasis**						
Control cohort	Reference	Reference	Reference	Reference	Reference	Reference
Asthma cohort without bronchiectasis	1.09 (1.03–1.14)	2.32 (2.03–2.65)	1.12 (1.06–1.17)	2.38 (2.08–2.72)	1.10 (1.05–1.16)	2.26 (1.98–2.59)
Asthma cohort with bronchiectasis	2.50 (2.07–3.02)	11.70 (8.47–16.18)	1.43 (1.18–1.73)	6.35 (4.59–8.78)	1.39 (1.15–1.68)	5.74 (4.15–7.95)
**Lung cancer**						
Control cohort	Reference	Reference	Reference	Reference	Reference	Reference
Asthma cohort without lung cancer	1.11 (1.06–1.16)	2.53 (2.22–2.87)	1.12 (1.07–1.18)	2.55 (2.24–2.90)	1.11 (1.05–1.16)	2.41 (2.11–2.74)
Asthma cohort with lung cancer	7.30 (4.60–11.58)	5.34 (0.75–37.94)	4.11 (2.59–6.52)	3.25 (0.46–23.09)	4.07 (2.57–6.46)	3.12 (0.44–22.10)

Data are presented as a ratio (95% CI).

Model 1: age and sex were adjusted; Model 2: age, sex, and type of insurance were adjusted.

HR, hazard ratio; CI, confidence interval; aHR, adjusted HR; COPD, chronic obstructive pulmonary disease.

Regardless of the presence or absence of bronchiectasis or lung cancer, asthmatic patients had an increased risk of overall mortality compared with the controls. The presence of bronchiectasis (aHR in Model 2 = 1.39, 95% CI = 1.15–1.68) or lung cancer (aHR in Model 2 = 4.07, 95% CI = 2.57–6.46) tended to further increase the risk of all-cause mortality in the asthmatic cohort compared with the control cohort (bronchiectasis in Fig. 3c and lung cancer in Fig. 3d).

Except for lung cancer, patients in the asthma cohort had a higher risk of respiratory mortality compared with subjects in the control cohort regardless of respiratory comorbidities. The presence of these respiratory comorbidities tended to further increase the risk of respiratory mortality in the asthmatic cohort compared with the control cohort (Table 2).

### Additive effect of asthma and its respiratory comorbidity on mortality

As shown in Table 3, asthma and respiratory comorbidities (COPD, pneumonia, and bronchiectasis) showed additive effects on respiratory mortality, but not on overall mortality. In contrast, asthma and lung cancer had an additive effect on overall mortality, but not on respiratory mortality.

**Table 3:** The risk of mortality according to the presence of asthma and its coexisting respiratory comorbidities.

	Unadjusted model		Model 1		Model 2	
	All-cause	Respiratory	All-cause	Respiratory	All-cause	Respiratory
	HR (95% CI)	HR (95% CI)	aHR (95% CI)	aHR (95% CI)	aHR (95% CI)	aHR (95% CI)
**COPD**						
Control cohort without COPD	Reference	Reference	Reference	Reference	Reference	Reference
Control cohort with COPD	2.91 (2.69–3.15)	6.48 (5.24–8.01)	1.25 (1.15–1.35)	2.48 (2.00–3.07)	1.24 (1.15–1.35)	2.42 (1.96–3.00)
Asthma cohort without COPD	0.80 (0.76–0.86)	1.41 (1.18–1.69)	0.97 (0.91–1.03)	1.76 (1.47–2.11)	0.96 (0.90–1.02)	1.70 (1.42–2.05)
Asthma cohort with COPD	3.11 (2.91–3.33)	10.90 (9.31–12.75)	1.52 (1.42–1.62)	4.78 (4.08–5.61)	1.48 (1.39–1.59)	4.42 (3.77–5.19)
**Pneumonia**						
Control cohort without pneumonia	Reference	Reference	Reference	Reference	Reference	Reference
Control cohort with pneumonia	2.84 (2.56–3.15)	5.29 (3.97–7.05)	1.75 (1.58–1.95)	3.03 (2.27–4.04)	1.75 (1.58–1.94)	3.01 (2.26–4.02)
Asthma cohort without pneumonia	1.02 (0.97–1.07)	2.12 (1.83–2.46)	1.05 (1.00–1.11)	2.20 (1.90–2.56)	1.04 (0.99–1.10)	2.11 (1.81–2.45)
Asthma cohort with pneumonia	1.98 (1.81–2.16)	6.36 (5.23–7.73)	1.62 (1.49–1.78)	5.01 (4.12–6.09)	1.59 (1.45–1.74)	4.60 (3.78–5.60)
**Bronchiectasis**						
Control cohort without BE	Reference	Reference	Reference	Reference	Reference	Reference
Control cohort with BE	3.01 (2.35–3.85)	8.88 (5.23–15.09)	1.42 (1.11–1.82)	3.82 (2.25–6.48)	1.41 (1.10–1.80)	3.69 (2.17–6.27)
Asthma cohort without bronchiectasis	1.09 (1.04–1.15)	2.37 (2.07–2.71)	1.12 (1.07–1.17)	2.42 (2.12–2.77)	1.10 (1.05–1.16)	2.30 (2.01–2.63)
Asthma cohort with bronchiectasis	2.52 (2.08–3.04)	11.95 (8.64–16.53)	1.43 (1.19–1.73)	6.45 (4.67–8.93)	1.40 (1.16–1.69)	5.86 (4.23–8.11)
**Lung cancer**						
Control cohort without lung cancer	Reference	Reference	Reference	Reference	Reference	Reference
Control cohort with lung cancer	8.68 (6.26–12.04)	12.89 (4.82–34.45)	4.02 (2.90–5.58)	5.68 (2.12–15.19)	3.95 (2.84–5.48)	5.32 (1.99–14.23)
Asthma cohort without lung cancer	1.11 (1.06–1.17)	2.54 (2.23–2.89)	1.13 (1.07–1.18)	2.56 (2.25–2.91)	1.11 (1.06–1.16)	2.42 (2.13–2.76)
Asthma cohort with lung cancer	7.32 (4.61–11.62)	5.37 (0.76–38.18)	4.12 (2.59–6.54)	3.27 (0.46–23.23)	4.08 (2.57–6.48)	3.13 (0.44–22.24)

Data are presented as a ratio (95% CI).

Model 1: age and sex were adjusted; Model 2: age, sex, and type of insurance were adjusted.

HR, hazard ratio; CI, confidence interval; aHR, adjusted HR; COPD, chronic obstructive pulmonary disease.

## Discussion

In this study, we compared the overall and respiratory mortality rates in patients with and without asthma using a large NHIS-NSC sample cohort. Patients with asthma had higher all-cause and respiratory mortality rates compared with subjects without asthma. The effects of asthma on all-cause mortality were more evident in the male subgroup, patients under medical aid, and subjects with COPD. Asthma had a more substantial effect on respiratory mortality in male subgroups and the younger population (age < 60 years). In addition, to the best of our knowledge, this is the first study in which higher mortality in asthmatics compared with non-asthmatics was comprehensively shown to be attributed to respiratory comorbidities other than COPD (pneumonia, bronchiectasis, and lung cancer). We also showed that asthma and lung cancer had an additive effect solely on overall mortality, asthma and other respiratory comorbidities had additive effects only on respiratory mortality in asthmatic patients.

A previous study evaluating 164,845 asthmatic patients and matched controls showed that all-cause mortality was approximately 1.25-fold higher in asthmatic patients compared with controls^4^. Our study confirmed these findings with a larger number of patients from a nationally representative database, showing that asthmatic patients had 1.13-fold higher mortality compared with subjects without asthma. These findings indicate that asthma management remains unsatisfactory, and proper asthma management strategies are needed, especially in males and the younger population. The reasons for high mortality in male asthmatics might be explained by a large gap in the smoking rate between Korean males and females^16^. Smoking is associated with severe asthmatic symptoms and exacerbations^17^. Smoking is also associated with increased risk of COPD and lung cancer in asthmatic patients^8,18^, which subsequently can increase mortality. The reasons why the effects of asthma on respiratory mortality are higher in the younger population remain unclear. One hypothesis is that the effects of asthma on mortality gradually decrease as patients age. Many elderly subjects have multiple comorbidities other than asthma that can lead to mortality^19^. Thus, the effects of asthma on mortality in the elderly might not be as high as in the younger population.

One of the critical findings of our study is that the higher mortality in asthmatic patients compared with subjects without asthma was mainly due to comorbid respiratory diseases. Smoking-related respiratory comorbidities, such as COPD and lung cancer, were the main risk factors for increased mortality in asthmatics compared with subjects without asthma. Regarding the impact of COPD on asthma, previous studies showed that COPD substantially increases the burden of symptoms, the rate of acute exacerbations, and the mortality rate in asthmatic patients^4,20–23^. Beyond this, our study provided informative data that the COPD-related increase of mortality in asthmatics was mostly respiratory-related, and there might be an additive effect of COPD and asthma on respiratory mortality. Another explanation for this phenomenon is the recent evidence linking asthma with the risk of lung cancer^8^. Our results showed that asthmatic patients with lung cancer had an extremely high risk of mortality compared with those without asthma. In addition, the risk of mortality was highest in asthmatic patients with lung cancer compared with those with other respiratory comorbidities. When we further analyzed the mortality according to the presence or absence of asthma and lung cancer, the risk of overall mortality in asthmatic patients with lung cancer was similar to that of non-asthmatics with lung cancer, suggesting that lung cancer itself determines the prognosis in asthmatics with lung cancer. Overall, the results indicate the need for strategies to prevent smoking-related pulmonary diseases, such as smoking cessation, in the management of asthmatic patients.

Bronchiectasis is a common comorbidity of asthma and is associated with severe symptoms and a larger number of exacerbations^7^. However, the effects of bronchiectasis on mortality in asthmatic patients have rarely been reported. A recent Spanish study evaluated the effects of in-hospital bronchiectasis mortality and found that in-hospital mortality was higher in asthmatic patients with bronchiectasis than in those without bronchiectasis (2.1% *vs*. 1.2%). However, the difference in mortality was not significant after adjusting for covariables^24^. In contrast, another study evaluated the impact of bronchiectasis on mortality in patients with severe asthma and showed that bronchiectasis was associated with all-cause mortality^25^. As the former study evaluated only in-hospital mortality, and the latter evaluated severe asthma patients, there are limited data on this issue. Showing the effects of bronchiectasis on increased all-cause and respiratory mortality and the additive effect of bronchiectasis on respiratory mortality in asthmatic patients using a nationwide large sample data is an important advantage of the present study.

Our study also showed that pneumonia is associated with an increased risk of long-term mortality in patients with asthma. Pneumonia caused a major increase in the risk of respiratory mortality, and its effect was additive to the impact of asthma on respiratory mortality. In line with our findings, the current guidelines including Korean asthma guidelines^26,27^ as well as the GINA guideline^28^ strongly recommend the preventive strategies of pneumonia using influenza vaccination and pneumococcal vaccination, which may be helpful to reduce pneumonia-related mortality. The guidelines also recommend stopping smoking in asthmatics to reduce the development of smoking-related diseases (e.g., COPD and lung cancer) as well as long-term mortality^26–28^. However, they do not provide opinions about the role of bronchiectasis on long-term mortality of mortality^26–28^. Currently, bronchiectasis is recommended to be considered as a differential diagnosis of asthma or as a comorbidity of severe asthma^26–28^. Given the additive impact of respiratory comorbidities on mortality in asthmatic patients, future guidelines may need to provide more space to address this topic. Our study results also warrant Korean government-led public health preventive actions to screen respiratory comorbidities and monitoring of the proper management of these diseases for patients with asthma.

Unexpectedly, we found that the number of patients using inhaled corticosteroid (ICS) was lower than should be. There might be some reasons for this phenomenon. The nationwide evaluation of all asthmatic patients might be associated with the inclusion of a relatively high proportion of subjects with mild asthma, in whom ICS use is low^29^. However, we would like to carefully suggest that this phenomenon was also likely to be caused by the low prescription of ICS in Korean patients with asthma, especially those treated in primary care^11,30^. Considering that low adherence to ICS is associated with increased risk of mortality in patients with asthma^31^, our study results suggest that an urgent strategy to increase ICS use is needed in Korea.

There are several limitations to our study. First, the NHIS-NSC database did not provide data on smoking history, body mass index, and pulmonary function, which might affect the mortality in patients with asthma. For example, the smoking rate in Korea is sex disproportional^32^. While 36.7% of Korean adult males were current smokers, 7.5% of Korean adult females were current smokers in 2018^33^. Thus, the increased mortality of asthma in males than in females might be attributed to the disproportional smoking rate by sex. Second, this study was performed in Korea. Although we analyzed a large number of patients using a nationally representative sample, the data might not apply to other ethnic groups in different countries. However, using the nationwide representative database is also an important advantage of the study. To the best of our knowledge, this is the first study that demonstrated increased mortality and causes of mortality in asthmatics compared with subjects without asthma using nationally representative data. Third, we did not adjust for compliance with medications. However, because different inhalation doses can be administered by one inhaler according to the asthma treatment protocol, it is complicated to analyze the medication usage ratio. In addition, some medications (e.g., budesonide/formoterol inhaler and oral corticosteroids) are used as controllers as well as relievers. Fourth, medial acid was twice as common in the asthma group than in the control group. It is hard to provide plausible reasons for this phenomenon with our study design. However, a possibility exists that asthma itself or comorbidities might have affected this since previous studies showed that low socioeconomic status, represented as medical aid in this study, is associated with asthma^34^ as well as other multiple comorbidities^35^. However, the impact of medical aid on our main result seems to be minimal as the adjustment of type of insurance did not significantly affect the results. Fifth, we did not consider the time-varying nature of asthma and respiratory comorbidities in our analyses, which were limited by the relatively small number of subgroup populations. Thus, future studies evaluating the impact of respiratory comorbidities on mortality in patients with asthma are needed to consider this issue. Lastly, the asthma cohort had more comorbidities compared to the control cohort, which made the evaluation of mortality more complex. Although the asthma cohort had a higher mortality than the control cohort, there is a possibility that some mutual risk factors for both asthma and other chronic diseases have influenced the results.

In conclusion, the mortality in patients with asthma was higher compared with subjects without asthma, especially in patients with pulmonary comorbidities, such as COPD, pneumonia, bronchiectasis, or lung cancer. While lung cancer showed an additive effect solely on overall mortality in patients with asthma, other respiratory comorbidities (COPD, pneumonia, and bronchiectasis) showed additive effects only on respiratory mortality in patients with asthma. Despite the recent advancement in asthma management, strategies to improve the mortality of asthma are still needed, especially in patients with pulmonary comorbidities.

## Supplementary Information

Supplementary Information.

## Data Availability

We used data from the National Health Insurance System (NHIS)-National Sample Cohort (NSC), a specialized data set provided by NHIS that includes a stratified random sample for the total eligible Korean population.

## Abbreviations

aHR: Adjusted hazard ratio
CI: Confidence interval
COPD: Chronic obstructive pulmonary disease
ICD-10: 10th revision of the International Statistical Classification of Diseases and Related Health Problems
NHIS-NSC: National Health Insurance System-National Sample Cohort
