# Supplementary material for: Additive effects of coexisting respiratory comorbidities on overall or respiratory mortality in patients with asthma: a national cohort study
Source: Sci Rep. 2022 May 16;12:8105. doi: 10.1038/s41598-022-12103-w (PMC9110422; doi:10.1038/s41598-022-12103-w)
Supplement: Supplementary file 1 — Supplementary Information. [file 41598_2022_12103_MOESM1_ESM.pdf]

Additive effects of Coexisting Respiratory Comorbidities on Overall or Respiratory Mortality in Patients with Asthma: A National Cohort Study

Short title: Increased mortality in asthma with pulmonary comorbidity

Yoomi Yeo[1*], Hyun Lee[1*], Jiin Ryu[2*], Sung Jun Chung[1], Tai Sun Park[1], Dong Won Park[1], Sang-Heon Kim[1], Tae Hyung Kim[1], Jang Won Sohn[1], Ho Joo Yoon[1], Kyung Hoon Min[3#], Ji-Yong Moon[1#]

[1]Department of Internal Medicine, Hanyang University College of Medicine, Seoul, Korea

[2]Biostatistical Consulting and Research Lab, Medical Research Collaborating Center, Hanyang University, Seoul, Korea

[3]Division of Respiratory and Critical Care Medicine, Department of Internal Medicine Korea University Guro Hospital, Korea University Medical School, Seoul, Korea

Corresponding author: Kyung Hoon Min, MD., Ph.D. and Ji-Yong Moon, M.D., Ph.D.

Supplemental TABLE 1. Baseline comorbidity profiles of the study population

	Total (n = 96,595)	Asthma cohort (n = 19,319)	Control cohort (n = 77,276)	p-value
**Respiratory diseases**	67,129 (69.5)	19,319 (100.0)	47,810 (61.9)	< 0.01
COPD	6,172 (6.4)	3,511 (18.2)	2,661 (3.4)	< 0.01
Pneumonia	4,374 (4.5)	2,866 (14.8)	1,508 (2.0)	< 0.01
Bronchiectasis	701 (0.7)	468 (2.4)	233 (0.3)	< 0.01
**Cardiovascular disease**	30,618 (31.7)	7,383 (38.2)	23,235 (30.1)	< 0.01
Hypertension	23,823 (24.7)	5,626 (29.1)	18,197 (23.6)	< 0.01
Angina	4,172 (4.3)	1,242 (6.4)	2,930 (3.8)	< 0.01
Myocardial infarction	632 (0.7)	203 (1.1)	429 (0.6)	< 0.01
Congestive heart failure	1,537 (1.6)	621 (3.2)	916 (1.2)	< 0.01
Cerebrovascular disease	4,489 (4.7)	1,059 (5.5)	3,430 (4.4)	< 0.01
**Endocrine diseases**	23,673 (24.5)	6,111 (31.6)	17,562 (22.7)	< 0.01
Diabetes mellitus	11,767 (12.2)	2,951 (15.3)	8,816 (11.4)	< 0.01
**Gastrointestinal diseases**	62,892 (65.1)	16,314 (84.5)	46,578 (60.3)	< 0.01
**Neurologic diseases**	16,788 (17.4)	4,723 (24.5)	12,065 (15.6)	< 0.01
**Mental and behavioral diseases**	16,500 (17.1)	4,641 (24.0)	11,859 (15.4)	< 0.01
**Musculoskeletal and connective tissue diseases**	50,811 (52.6)	12,546 (64.9)	38,265 (49.5)	< 0.01
Osteoporosis	7,141 (7.4)	1,762 (9.1)	5,379 (7.0)	< 0.001
**Charlson Comorbidity Index***	0 (0-1)	0 (0-1)	0 (0-1)	< 0.001

Data are presented as numbers (%).
*Asthma was not considered as respiratory comorbidity for the calculation of the Charlson Comorbidity Index since we hypothesized the impact of asthma

on mortality was unknown.
COPD, chronic obstructive pulmonary disease.

Supplemental FIGURE 1. Flow chart of the study participants.

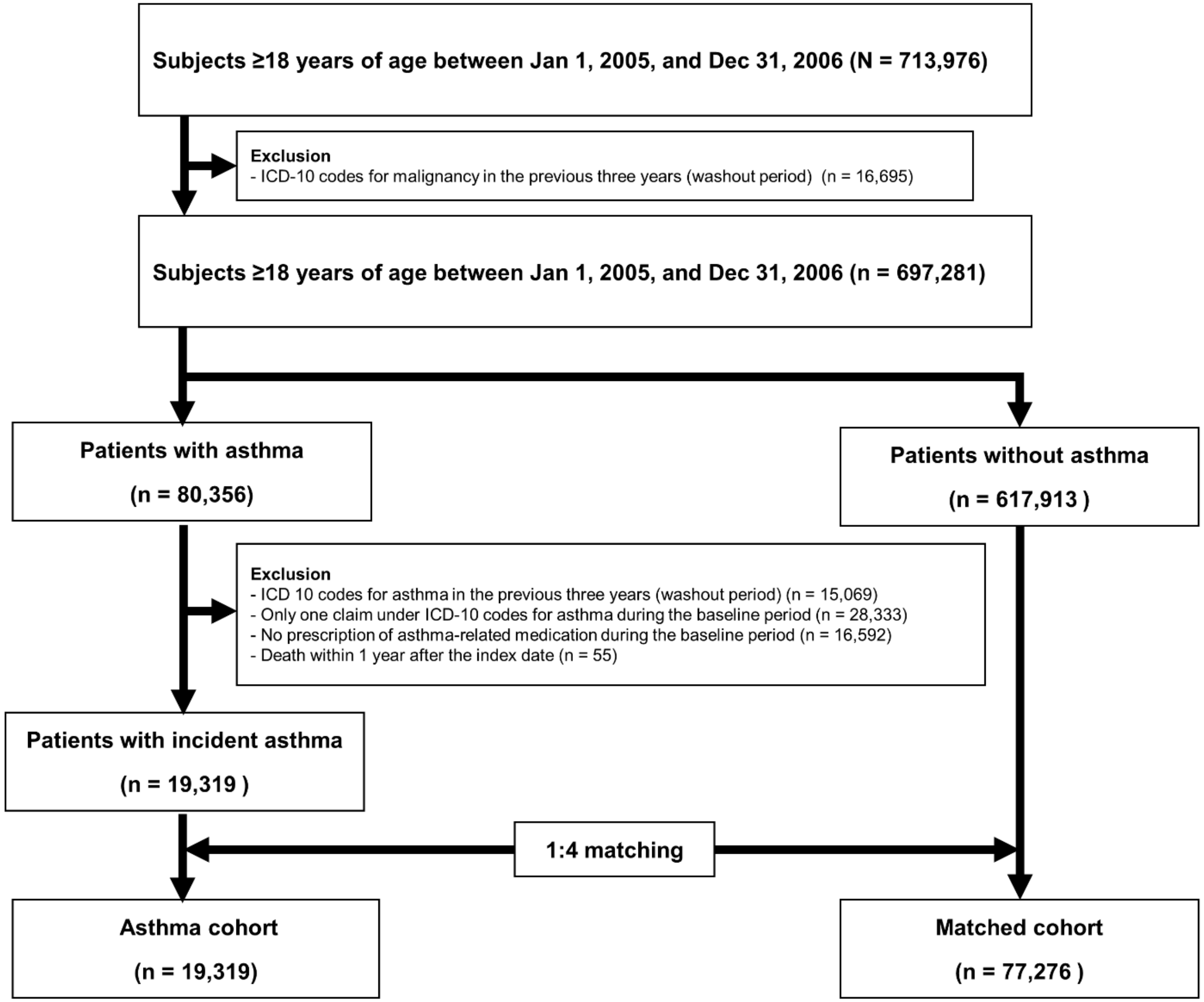

Adult asthma was defined based on the following criteria: (1) 18 years of age or older, (2) at least two claims under the 10th revision of the International

Statistical Classification of Diseases and Related Health Problems (ICD-10) codes J45–46, and (3) at least one claim for prescription of asthma-related drugs including inhaled or systemic corticosteroids, bronchodilators, leukotriene receptor antagonists, or xanthine derivatives.
